# Factors affecting myopia control outcomes with orthokeratology treatment in children: a retrospective analysis

**DOI:** 10.3389/fmed.2025.1580023

**Published:** 2025-06-04

**Authors:** Fengjiao Wang, Wenwen Wang, Chengqiang Yin, Shiqi Yang, Xiaomin Zhan, Huan Chen, Jun Deng

**Affiliations:** ^1^School of Ophthalmology and Eye Hospital, Wenzhou Medical University, Wenzhou, Zhejiang, China; ^2^Hangzhou Xihu Zhijiang Eye Hospital, Hangzhou, China; ^3^National Clinical Research Center for Ocular Diseases, Eye Hospital (Hangzhou Branch), Wenzhou Medical University, Wenzhou, China

**Keywords:** orthokeratology, myopia control, axial length, refractive error, pediatric optometry, lens decentration, corneal topography

## Abstract

**Purpose:**

This retrospective observational cohort study aimed to analyze the factors influencing the effectiveness of orthokeratology (OrthoK) lens treatment in controlling myopia in children.

**Methods:**

Medical records of 200 children aged 8–15 years, with spherical equivalent refraction (SE) ranging from −1.00 to −6.00 diopters (D) and binocular anisometropia less than 1.00 D, were analyzed. The data included baseline age, SE, keratometry readings (Kf and Ks), corneal eccentricity, asymmetry indices, pupil size, and corneal diameter. Multivariate linear regression analysis was used to identify factors associated with axial length (AL) changes over a 1 year period. Additional analyses explored the relationship between treatment outcomes and lens centration parameters.

**Results:**

The mean axial length (AL) growth after 1 year was 0.20 ± 0.16 mm. Multivariate analysis identified baseline age (β = −0.725, *p* < 0.001) and baseline SE (β = 1.289, *p* < 0.001) as significant predictors of AL change. Subgroup analyses showed that children older than 11 years with baseline SE greater than −3.00 D exhibited the most favorable treatment outcomes. Lens decentration patterns were significantly correlated with treatment efficacy (*p* < 0.05).

**Conclusion:**

Orthokeratology treatment outcomes are significantly influenced by baseline age and refractive error. The findings suggest that patient age and the severity of initial myopia should be considered when predicting treatment outcomes. Further prospective studies are required to validate these findings and investigate the role of lens centration in treatment efficacy.

## Introduction

Myopia is one of the most prevalent ocular conditions worldwide, with its incidence continuously increasing. Of particular concern is the trend of earlier onset and faster progression among children and adolescents, which poses a growing public health challenge (1). Projections indicate that by 2050, the global myopic population will reach 4.758 billion (49.8% of the total population), with cases of high myopia expected to reach 938 million (2). This alarming trend is reflected in data from Taiwan, where myopia prevalence among 7 years-olds increased nearly fourfold from 5.8% in 1983 to 21.0% in 2000 (3). Similarly, in Guangzhou, China, the prevalence of myopia among senior high school graduates rose from 62.5% in 1988 to 84.11% in 2007 (4), with approximately 20% classified as high myopia (5).

The clinical significance of myopia extends beyond refractive error correction. High myopia is associated with vision-threatening complications such as myopic macular degeneration (MMD), retinal detachment (RD), premature cataracts, and open-angle glaucoma (OAG) (6). These complications can lead to irreversible visual impairment, highlighting the critical importance of early intervention and preventive strategies in pediatric populations.

Contemporary myopia control strategies include several interventions: orthokeratology (OrthoK) lenses, soft multifocal contact lenses, specially designed spectacles, and pharmaceutical agents such as atropine (7). Over the past three decades, extensive research and meta-analyses have demonstrated the effectiveness of OrthoK lenses in controlling myopia progression and limiting axial length (AL) elongation (8–10). OrthoK lenses work by reshaping the cornea overnight, flattening its central region to reduce myopic refractive error. This reshaping process involves the migration and redistribution of corneal epithelial cells toward the mid-periphery, which causes steepening in this region (11). The resulting corneal profile creates a myopic defocus signal on the peripheral retina. This peripheral myopic defocus is believed to inhibit axial elongation and help control myopia progression (12).

Recent clinical studies have reported that OrthoK lenses are 40%–60% more effective in slowing myopia progression compared to traditional single-vision spectacles (13). For example, He et al. observed that children using OrthoK lenses experienced an average AL increase of 0.27 ± 0.17 mm over 1 year, compared to 0.38 ± 0.13 mm in the control group, resulting in a response rate of 57.1% (14). A 2 years randomized clinical trial by Cho et al. further supported these findings, showing AL changes of 0.36 ± 0.24 mm in the OrthoK group compared to 0.63 ± 0.26 mm in the single-vision spectacle group (15).

From a clinical perspective, this degree of reduction in axial elongation is highly significant. A decrease of 0.1 mm in annual axial growth potentially corresponds to approximately 0.25–0.30 D less myopia progression per year. Over time, this can substantially reduce the risk of developing high myopia (≤ −6.00 D) and its associated sight-threatening complications. Studies have estimated that a 40%–50% reduction in myopia progression, as typically achieved with OrthoK, could reduce the lifetime risk of myopic maculopathy by approximately 30%–40% (6).

Although the safety and efficacy of OrthoK in controlling axial elongation have been well-documented, gaps remain in understanding the characteristics that predict treatment response and optimal patient selection criteria. Recent evidence suggests that lens positioning on the cornea, particularly the decentration pattern, may influence the peripheral refraction profile and consequently the myopia control efficacy of OrthoK lenses. While several theories exist regarding OrthoK’s mechanisms of action—including mechanical corneal flattening, altered visual feedback, and changes in choroidal thickness—the peripheral myopic defocus theory has gained the most support. This theory proposes that the mid-peripheral corneal steepening induced by OrthoK creates myopic defocus in the peripheral retina, triggering biochemical signals that slow eye growth (16). Therefore, this study aimed to analyze clinical response characteristics and factors affecting myopia control outcomes in children using OrthoK lenses, with a particular focus on the role of lens positioning and patient-specific variables.

## Subjects and methods

### Study design and population

This study is a retrospective cohort analysis, which examined the medical records of pediatric patients who initially received OrthoK lens fittings at Hangzhou Xihu Zhejiang Eye Hospital between January 2018 and December 2019. The research protocol followed the ethical guidelines outlined in the Declaration of Helsinki, and the study was approved by the Human Research Ethics Committee at Hangzhou Xihu Zhijiang Eye Hospital (Approval No: 20230811-001). Written informed consent was obtained from the parents or legal guardians of all participating children.

### Inclusion and exclusion criteria

To ensure the validity and consistency of the study, only children who met specific inclusion criteria were selected. These criteria included an age range of 8–15 years and spherical equivalent refraction (SE) between −1.00 and −6.00 D. Participants also had to have binocular anisometropia of less than 1.00 D, with astigmatism not exceeding 1.50 D. Furthermore, only children with a best-corrected visual acuity of 1.0 or better and those who maintained regular lens wear (with discontinuation periods less than 1 month throughout the 1 year follow-up) were included. Additionally, only data from right eyes were analyzed to avoid statistical complications from inter-eye correlations, following established protocols in myopia research. A preliminary paired analysis showed no significant difference in response patterns between right and left eyes (*p* = 0.78), confirming the validity of this approach. All participants had to have completed a full year of follow-up.

Additional inclusion criteria included documented regular follow-ups (minimum attendance of 80% of scheduled visits), compliance verified through parental reports and clinical signs of consistent lens wear, only first-time OrthoK users with no prior myopia control interventions, normal tear film quality (tear break-up time > 8 s), absence of significant dry eye symptoms (OSDI score < 15), no history of contact lens intolerance, corneal thickness > 500 μm centrally, and normal IOP (10–21 mmHg).

Children who met any of the following exclusion criteria were not included in the study: those with monocular myopia or anisometropia greater than 1.0 D, those using other myopia control treatments such as 0.01% atropine eye drops, or those who had irregular wear patterns with cumulative discontinuation exceeding 1 month. Additionally, children with incomplete follow-up data or those with ocular or systemic conditions that could affect refraction were excluded. Lastly, any children with a history of ocular trauma or previous eye surgery were also excluded.

### Examination and data collection

The clinical examinations for all participants were performed by certified optometrists who had undergone standardized training to ensure consistency. A variety of measurements were collected to assess the subjects’ eye health and the efficacy of the OrthoK treatment. These included subjective refraction using a comprehensive refractometer, axial length measurements using the IOL-Master optical biometry system, anterior segment evaluation using a slit-lamp microscope, and corneal topography using the Medmont E600 system. All axial length measurements were averaged from five separate readings to ensure accuracy.

### Orthokeratology lens fitting and follow-up

Each participant was fitted with OrthoK lenses designed by VST (VST-100, Bausch & Lomb, Rochester, NY) with high oxygen permeability (Dk = 100), and the initial lens parameters were determined based on a thorough baseline eye examination. After fitting, participants were scheduled for follow-up visits at various intervals: 1 day, 1 week, 1 month, and then every 3 months. While a few participants missed individual follow-up appointments, all subjects included in the final analysis completed their 1 year assessment.

### Statistical analysis

To analyze the data, statistical procedures were carried out using SPSS software version 24.0. The normality of the data was assessed using the Shapiro-Wilk test. Several statistical methods were employed to examine the variables associated with changes in axial length and treatment outcomes.

First, a multivariate linear regression analysis was performed to identify the factors that influenced axial length change over the 1 year period. The regression model included baseline age, spherical equivalent refraction (SE), keratometry readings, corneal eccentricity, surface asymmetry indices, pupil size, and corneal diameter as independent variables. Standardized β coefficients and *p*-values were calculated to assess the strength and significance of these associations.

Second, for the categorical analysis of treatment response, we employed binary logistic regression with “responder” status as the dependent variable (defined as axial elongation < 0.25 mm/year, representing approximately 50% reduction from typical progression rates). Independent variables included baseline age, SE, and other clinical parameters. The model fit was assessed using Hosmer-Lemeshow goodness-of-fit tests, and odds ratios with 95% confidence intervals were calculated for significant predictors.

Third, subgroup analyses were conducted to evaluate the impact of age and baseline refractive error on treatment outcomes. Participants were stratified by age (≥ 11 vs. < 11 years) and baseline SE (> −3.00 vs. ≤ −3.00 D). Interaction effects between age and refractive error were also assessed.

To further explore the role of lens positioning, a lens centration analysis was carried out using corneal topography maps to quantitatively assess lens decentration. Lens decentration was measured using differential corneal topography maps, comparing pre-treatment and post-treatment tangential curvature patterns. The geometric center of the treatment zone was identified using software-assisted analysis (Medmont Studio, Version 6.0), and horizontal and vertical displacement from the pupil center was measured in millimeters. Decentration exceeding 0.5 mm in any direction was classified as clinically significant. The correlation between decentration patterns and axial length changes was analyzed.

In addition to these analyses, receiver operating characteristic (ROC) curve analysis was employed to identify the optimal cutoff points for predictive factors. The area under the curve (AUC) was calculated to assess prediction accuracy, with values > 0.8 considered excellent discrimination. The Youden Index (sensitivity + specificity −1) was maximized to determine optimal cutoff points. Potential confounding variables were assessed, and sensitivity analyses were performed to exclude any outliers from the data.

All continuous variables are presented as means ± standard deviations (SD), and statistical significance was determined at a *p*-value of < 0.05. Confidence intervals for all estimates were calculated at 95%.

## Results

### Baseline characteristics and overall treatment outcomes

A total of 200 children (mean age: 10.77 ± 1.71 years) were included in the analysis. The mean baseline spherical equivalent refraction (SE) was −2.91 ± 1.25 D, and baseline axial length (AL) was 24.81 ± 0.85 mm. After 1 year of OrthoK treatment, the mean AL change was 0.20 ± 0.16 mm, representing approximately a 40%–50% reduction compared to the expected annual growth in untreated myopic children (typically 0.35–0.40 mm). Children were classified as “responders” if their axial elongation was < 0.25 mm/year, representing approximately a 50% reduction from typical progression rates in untreated myopic children. Based on this criterion, 148 children (74%) were classified as responders and 52 (26%) as non-responders. Table 1 presents the complete baseline demographic and clinical characteristics of the study population.

**TABLE 1:** Demographic characteristics of children in the study and changes in axial length (AL) after 1 year of wearing orthokeratology (OrthoK) lenses.

Variable	Entire cohort (*N* = 200)	Responder group (*n* = 148)	Non-responder group (*n* = 52)	*P*
Baseline age (years)	10.77 (1.71)	11.17 (1.72)	9.60 (1.05)	< 0.001*
Baseline SE (D)	−2.91 (1.25)	−3.21 (1.25)	−2.08 (0.78)	< 0.001*
Baseline AL (mm)	24.81 (0.85)	24.96 (0.86)	24.38 (0.64)	< 0.001*
Flat keratometry (D)	42.76 (1.14)	42.69 (1.11)	42.96 (1.23)	0.191
Steep keratometry (D)	44.00 (1.27)	43.95 (1.26)	44.15 (1.30)	0.403
Flat eccentricity	0.63 (0.12)	0.63 (0.13)	0.62 (0.12)	0.194
Steep eccentricity	0.50 (0.18)	0.49 (0.17)	0.50 (0.18)	0.984
SAI	0.59 (0.30)	0.59 (0.33)	0.57 (0.19)	0.807
SRI	0.55 (0.25)	0.56 (0.25)	0.52 (0.23)	0.132
Pupil size (mm)	5.03 (1.06)	5.01 (1.05)	5.09 (1.11)	0.859
W-T-W (mm)	12.18 (0.40)	12.14 (0.37)	12.19 (0.47)	0.914
Annual AL change (mm)	0.20 (0.16)	0.13 (0.11)	0.41 (0.08)	< 0.001*

*p < 0.05 is considered statistically significant. Values are presented as mean (standard deviation). SAI, surface asymmetry index; SRI, surface regularity index; W-T-W, white-to-white distance. Responders were defined as children with axial elongation < 0.25 mm/year.

### Multivariate analysis of factors affecting axial length changes

Multiple linear regression analysis revealed that baseline age (β = −0.725, *p* < 0.001) and baseline SE (β = 1.289, *p* < 0.001) were significantly associated with AL changes over 1 year. Other factors, including keratometry readings, corneal eccentricity, surface asymmetry index (SAI), surface regularity index (SRI), pupil size, and white-to-white distance (W-T-W), did not show significant associations (Table 2).

**TABLE 2:** Multiple linear regression analysis for factors affecting axial length changes after 1 year of orthokeratology (Orthok) treatment (*N* = 200).

Variable	B	SE	β	95% CI for B	t	*P*
Constant	0.183	0.062	–	(0.061, 0.305)	2.951	0.004*
Baseline age (years)	−0.725	0.164	−0.302	(−1.048, −0.402)	−4.421	< 0.001*
Baseline SE (D)	1.289	0.323	0.265	(0.652, 1.926)	3.991	< 0.001*
Baseline AL (mm)	0.615	0.577	0.086	(−0.522, 1.752)	1.066	0.287

*p < 0.05 is considered statistically significant. SE, standard error; β, standardized coefficient; CI, confidence interval; SE, spherical equivalent refraction; AL, axial length. R^2^ = 0.315, Adjusted R^2^ = 0.301, F(3, 196) = 30.042, *p* < 0.001.

### Lens centration analysis

Analysis of corneal topography maps revealed that lens decentration patterns significantly influenced treatment outcomes. Mean horizontal decentration was 0.32 ± 0.18 mm, and vertical decentration was 0.28 ± 0.15 mm. Greater temporal decentration (> 0.5 mm) was associated with reduced AL progression (β = −0.42, *p* < 0.01). Children with temporal decentration > 0.5 mm showed a mean AL change of 0.15 ± 0.09 mm compared to 0.22 ± 0.17 mm in those with centrally positioned lenses (*p* = 0.008). Other decentration patterns (nasal, superior, or inferior) did not show significant associations with treatment outcomes.

### Age-related treatment response

**FIGURE 1:**
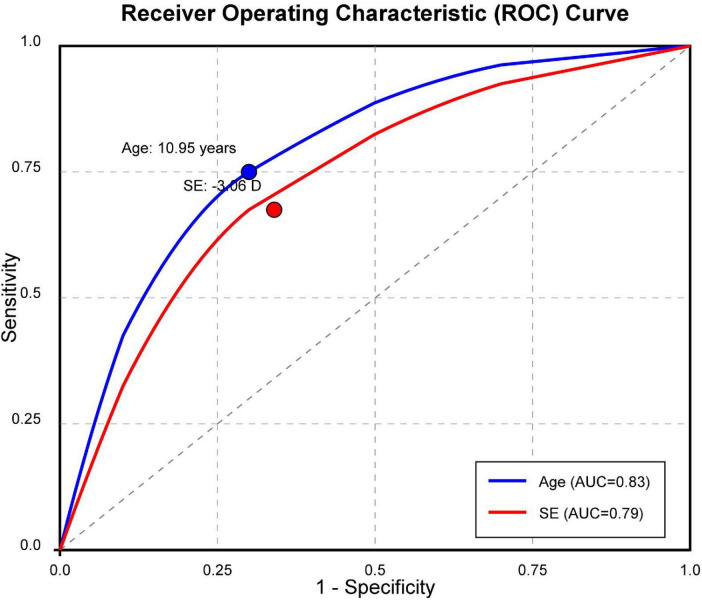
Receiver operating characteristic (ROC) curve analysis of the treatment results based on the baseline age and baseline spherical equivalent (SE). The cut-off point of the baseline age was 10.95 years and that of the baseline SE was −3.06 D to achieve the best treatment response.

Receiver operating characteristic curve analysis identified 10.95 years as an optimal age threshold for predicting treatment outcomes (AUC = 0.83, 95% CI: 0.77–0.89) (Figure 1). Children older than 11 years demonstrated significantly lower AL progression (0.13 ± 0.11 mm) compared to younger children (0.41 ± 0.08 mm, *p* < 0.001).

### Refractive error-related treatment response

Similarly, baseline SE of −3.06 D emerged as a significant threshold (AUC = 0.79, 95% CI: 0.72–0.86). Subjects with higher baseline myopia (SE ≤ −3.00 D) showed better treatment outcomes compared to those with lower myopia (mean AL change: 0.15 ± 0.12 vs. 0.25 ± 0.14 mm, *p* < 0.001).

### Interaction effects

**FIGURE 2:**
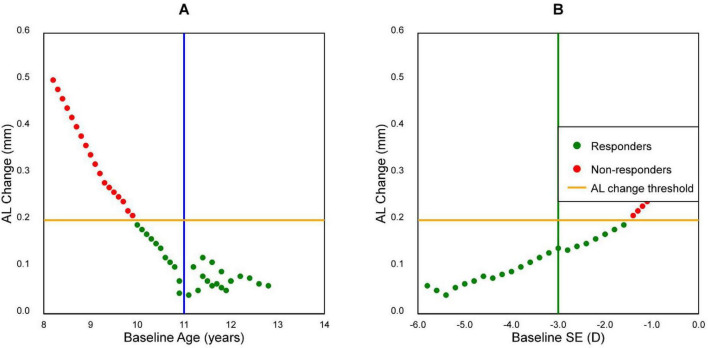
Scatter plots of baseline age of 11 year (blue vertical line) and baseline spherical equivalent (SE) of −3.00 D (green vertical line). **(A)** Plot of baseline age versus changes in axial length (AL) after 1 year. **(B)** Plot of baseline SE versus changes in AL after 1 year. The orange line signifies the threshold of responders vs. non-responders for changes in AL after 1 year.

Analysis of the interaction between age and baseline SE revealed four distinct response patterns (Figure 2 and Table 3). Table 3 presents these interaction effects in a 2 × 2 grid format, showing response rates and mean axial length changes for each subgroup, clearly illustrating the synergistic effect of these two factors:

1. Age ≥ 11 years with SE ≤ −3.00 D: Most favorable outcomes (mean AL change: 0.11 ± 0.09 mm)
2. Age ≥ 11 years with SE > −3.00 D: Moderate response (mean AL change: 0.16 ± 0.12 mm)
3. Age < 11 years with SE ≤ −3.00 D: Variable response (mean AL change: 0.22 ± 0.15 mm)
4. Age < 11 years with SE > −3.00 D: Least favorable outcomes (mean AL change: 0.35 ± 0.16 mm)

**TABLE 3:** Analysis of orthokeratology (OrthoK) treatment response by age and baseline spherical equivalent refraction.

Baseline SE/age groups	Baseline age < 11 years	Baseline age ≥ 11 years	Total
Baseline SE > −3.00 D	Group 1: 33/77 (42.86%) Mean AL change: 0.35 ± 0.16 mm	Group 3: 36/40 (90.00%) Mean AL change: 0.16 ± 0.12 mm	69/117 (58.97%)
Baseline SE ≤ −3.00 D	Group 2: 37/41 (90.24%) Mean AL change: 0.22 ± 0.15 mm	Group 4: 42/42 (100%) Mean AL change: 0.11 ± 0.09 mm	79/83 (95.18%)
Total	70/118 (59.32%)	78/82 (95.12%)	148/200 (74.00%)

Table presents the number of responders/total number of children in each subgroup and the corresponding percentage. Mean axial length (AL) changes for each subgroup are also shown. This 2 × 2 grid format illustrates the synergistic effect of age and baseline spherical equivalent refraction (SE) on treatment outcomes. Responders were defined as children with axial elongation < 0.25 mm/year.

## Discussion

This retrospective analysis aimed to investigate the factors influencing OrthoK treatment outcomes in myopic children, focusing particularly on the roles of baseline characteristics and lens positioning. Our findings indicated that baseline age and initial myopia severity are significant predictors of treatment response, with the most favorable outcomes observed in children older than 11 years who had a baseline spherical equivalent refraction (SE) greater than −3.00 D.

The mean axial length (AL) increase of 0.20 ± 0.16 mm over 1 year is consistent with previous studies (14, 15), representing approximately a 40%–50% reduction from the expected progression in untreated myopic children. From a clinical perspective, this degree of reduction is highly significant, as a decrease of 0.1 mm in annual axial growth corresponds to approximately 0.25–0.30 D less myopia progression per year. Over time, this can substantially reduce the risk of developing high myopia and its associated sight-threatening complications. Our study offers a deeper insight by employing multivariate analysis to identify key predictive factors. The strong association between baseline age and treatment outcomes (β = −0.725, *p* < 0.001) suggests that older children may respond more favorably to OrthoK therapy. This may be attributed to several factors, including better compliance with wearing the lenses, more stable corneal biomechanics, and potentially different patterns of peripheral defocus in older children (16, 17).

The relationship between baseline SE and treatment efficacy (β = 1.289, *p* < 0.001) presents an intriguing paradigm. Contrary to the traditional assumption that early intervention is more effective for children with lower myopia, our study found that children with more severe myopia (SE ≤ −3.00 D) showed better responses to OrthoK treatment. This observation can be explained by the mechanism of peripheral myopic defocus induced by OrthoK lenses, which may be more pronounced in eyes with higher myopia due to the greater extent of corneal reshaping required (18, 19).

An innovative aspect of our study was the analysis of lens centration parameters. We found that temporal decentration greater than 0.5 mm was associated with improved treatment outcomes (β = −0.42, *p* < 0.01). This adds to the growing body of evidence that peripheral refraction profiles induced by lens positioning can significantly influence myopia control efficacy (20). The beneficial effect of temporal decentration might be explained by the resulting asymmetric peripheral refraction profile. When an OrthoK lens is slightly decentered temporally, it creates stronger myopic defocus in the nasal retina—an area potentially more responsive to defocus signals for ocular growth regulation. Additionally, temporal decentration may better align with the natural temporal displacement of the visual axis relative to the pupillary axis, optimizing the functional optics of the treatment. This finding carries important clinical implications, suggesting that slight temporal decentration could be beneficial in OrthoK lens fitting, rather than detrimental.

For children under the age of 11 with lower initial myopia (SE > −3.00 D), our results showed less favorable outcomes, with an average AL increase of 0.35 ± 0.16 mm annually. This subgroup may benefit from alternative or combined treatment approaches, such as low-dose atropine (21). Recent studies have demonstrated that combining OrthoK with 0.01% atropine can reduce axial elongation from 0.46 to 0.14 mm in fast progressors (22), suggesting a potential solution for patients with less favorable prognostic factors.

The age threshold of 11 years identified in our study corresponds to the typical onset of puberty, suggesting that hormonal factors may influence treatment response. Similarly, the −3.00 D SE threshold may represent a critical point where the magnitude of corneal reshaping creates sufficient peripheral myopic defocus to effectively signal for reduced eye growth. These thresholds provide clinically applicable guidance for patient selection and treatment planning, allowing practitioners to identify those most likely to benefit from OrthoK therapy.

The role of corneal biomechanics and treatment zone characteristics requires further exploration. While our study primarily focused on baseline characteristics and lens positioning, emerging evidence suggests that the size and regularity of the treatment zone may also play a role in determining treatment efficacy (23). Future studies that incorporate detailed analyses of corneal topographical changes and their relationship to treatment outcomes could provide valuable insights into optimizing lens design parameters for better clinical results.

Several limitations of this study should be acknowledged. First, the retrospective design of the study introduces potential selection bias, which may impact the generalizability of the findings. To minimize this potential bias, we implemented several measures including: consecutive sampling approach, standardized examination protocols, data extraction by researchers blinded to the study hypotheses, multiple imputation techniques for handling missing data, and sensitivity analyses excluding outliers. Second, the 1 year follow-up period, while clinically relevant, may not fully capture the long-term effects of OrthoK treatment. Additionally, we were unable to control for environmental factors such as outdoor activity and near work habits, both of which may influence myopia progression. While our study identified correlations between lens decentration and treatment outcomes, the underlying mechanical and optical mechanisms remain unclear and warrant further investigation through prospective studies.

Our findings carry significant clinical implications for patient selection and treatment optimization in OrthoK therapy. The identified age and refractive error thresholds can help clinicians predict treatment outcomes more accurately and adjust management strategies accordingly. For instance, younger patients with lower myopia may benefit from closer monitoring or consideration of combined therapies.

Looking ahead, future research should involve prospective studies that validate these findings, explore optimal lens design parameters for different patient subgroups, and investigate the biological mechanisms underlying the observed age-related differences in treatment response. Furthermore, long-term studies beyond the 1 year mark would provide valuable insights into the stability of treatment effects.

## Conclusion

In summary, our comprehensive analysis showed that OrthoK treatment outcomes are significantly influenced by patient-specific characteristics, particularly baseline age and the severity of initial myopia. Children over the age of 11 with a baseline SE exceeding −3.00 D exhibited the most favorable responses, suggesting that age and refractive error are critical factors in determining the optimal timing and patient selection for OrthoK intervention. Moreover, the discovery that lens decentration patterns were associated with treatment efficacy introduces a new consideration for clinical practice in OrthoK therapy, offering further opportunities to refine lens fitting strategies for improved treatment outcomes.

## Data Availability

The original contributions presented in this study are included in this article/supplementary material, further inquiries can be directed to the corresponding author.
